# The regulatory role of antisense lncRNAs in cancer

**DOI:** 10.1186/s12935-021-02168-4

**Published:** 2021-08-30

**Authors:** Biao Liu, Wei Xiang, Jiahao Liu, Jin Tang, Jinrong Wang, Bin Liu, Zhi Long, Long Wang, Guangming Yin, Jianye Liu

**Affiliations:** Department of Urology, The Third Xiangya Hospital of Central South University, No.138, Tongzipo Road, Changsha, 410013 Hunan China

**Keywords:** Antisense lncRNA, Cancer, Transcriptional modulation, Translational control, Target therapy

## Abstract

Antisense long non-coding RNAs (antisense lncRNAs), transcribed from the opposite strand of genes with either protein coding or non-coding function, were reported recently to play a crucial role in the process of tumor onset and development. Functionally, antisense lncRNAs either promote or suppress cancer cell proliferation, migration, invasion, and chemoradiosensitivity. Mechanistically, they exert their regulatory functions through epigenetic, transcriptional, post-transcriptional, and translational modulations. Simultaneously, because of nucleotide sequence complementarity, antisense lncRNAs have a special role on its corresponding sense gene. We highlight the functions and molecular mechanisms of antisense lncRNAs in cancer tumorigenesis and progression. We also discuss the potential of antisense lncRNAs to become cancer diagnostic biomarkers and targets for tumor treatment.

## Background

**Fig. 1:**
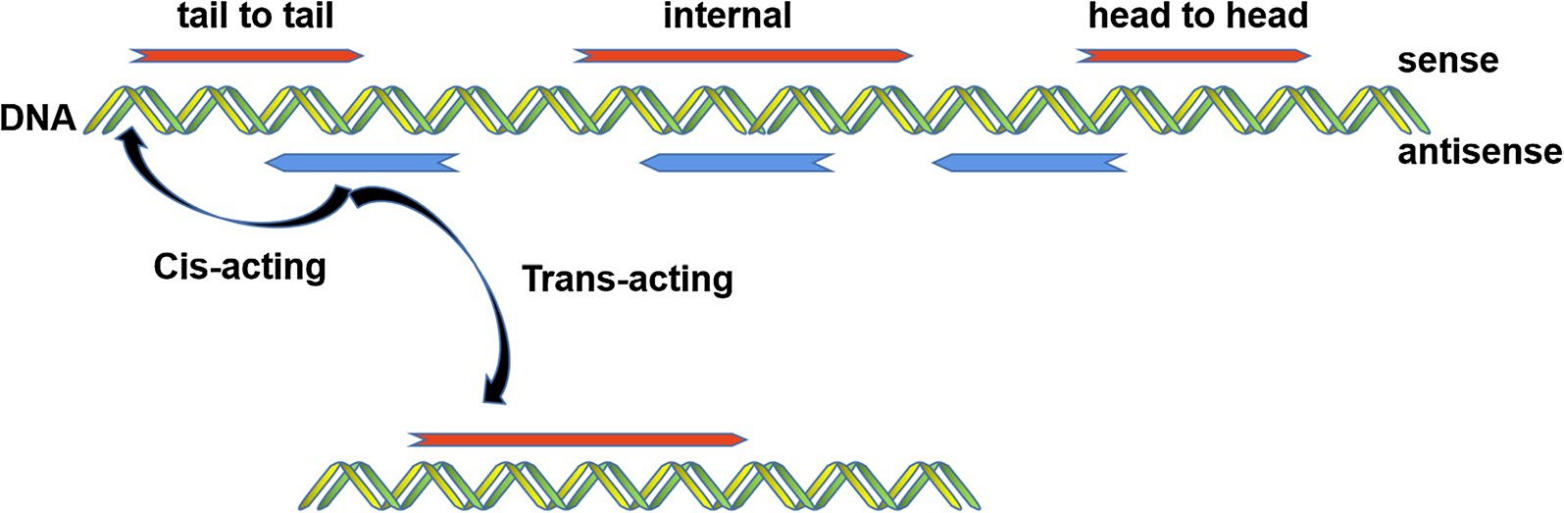
According to the original location in relative to its sense transcripts, antisense lncRNA can be further divided to three groups, tail to tail (two transcripts overlap in the 3ʹregion), internal (antisense lncRNA is completely covered by its sense transcripts) and head to head (two transcripts overlap in the 5ʹregion)

Protein-coding sequences account for less than 2% of the human genome, whereas most of the remaining regions of both DNA strands have the ability to be transcribed into RNAs. These RNAs cannot be translated into proteins, and are thus termed non-coding RNAs (ncRNAs) [1–3]. NcRNAs were previously considered as non-functional molecules [4]; however, recently, increasing evidence indicates that ncRNAs play an important role in regulating the expression of proteins and modulating various biological processes [5]. Based on their length, ncRNAs are classified into two types, and those RNA molecules that are more than 200 nucleotides are defined as long non-coding RNAs (lncRNAs). Accordingly, the other type is categorized as small non-coding RNAs [6, 7]. LncRNAs can be further classified into several groups, a large proportion of which are antisense lncRNAs, other groups include intergenic lncRNAs, intronic lncRNAs, and bidirectional (or divergent) lncRNAs [8–10]. Antisense lncRNAs are transcribed from the opposite strand of genes which have protein-coding or non-coding function (Fig. 1) [11]. They are defined according to the nearest protein-coding gene position, the same as ncRNAs, and they have no ability to be translated into proteins. Antisense lncRNAs are differentially expressed across different cell types, and regulate the expression of specific genes to modulate different signaling pathways [12]. Interestingly, they can exert their role through cis or trans regulation. Cis-acting antisense lncRNAs modulate the expression of the genes from which they originated by interacting with the promoter region with perfect sequence complementarity, while trans-acting antisense lncRNAs, through imperfect sequence complementarity, affect the expression of other genes [13]. In this review, we will elaborate the roles and molecular mechanisms of antisense lncRNAs in the process of tumorigenesis and tumor progression.

## Antisense LncRNAs and cancer

Cancer is regarded as a genetic disease, in which mutations of protooncogenes or cancer suppressor genes result in uncontrolled cell growth [14]. Recently, antisense lncRNAs were reported to modulate the expression of genes that play an important role in tumorigenesis and cancer progression [15, 16]. Dysregulation of antisense lncRNAs has been observed in almost all types of cancer, acting as tumor promoters or suppressors. For example, overexpression of *CDKN2B-AS1* in hepatocellular carcinoma (HCC) cells promotes tumor growth and metastasis [17]. In non-small cell lung cancer (NSCLC) tissues and cell lines, upregulated *NNT-AS1* promotes the proliferation and invasion of cancer cells [18]. In addition, the level of *VPS9D1-AS1* is negatively associated with tumor progression and poor prognosis in gastric cancer (GC) [19]. Reduced expression of *B3GALT5-AS1* in colon cancer tissues results in cancer cell migration and invasion [20]. There are many ways to identify antisense lncRNAs. RNA sequencing (RNA-seq) analysis is commonly used method to investigate the transcriptome profile of lncRNAs. By quantifiably detecting lncRNAs, upregulated or downregulated lncRNAs can be identified. This method has contributed significantly to the study of antisense lncRNAs [21]. For example, using RNA-seq data from 60 samples collected from 20 patients with HCC, Yang et al. newly assembled 8,603 lncRNAs, 16% of which were antisense lncRNAs. The authors found that antisense lncRNA *HAND2-AS1* was the only downregulated lncRNA in portal vein tumor thrombosis (PVTT), suggesting that *HAND2-AS1* is associated with cancer metastasis [22]. Antisense lncRNAs do not encode protein, and in most cases, they function upstream of various signaling pathways. Mechanistic investigations revealed that antisense lncRNAs can affect biological process in both the nucleus and cytoplasm, such as epigenetic modulations and translational control. Furthermore, the aberrant expression of antisense lncRNAs is responsible for chemoradioresistance, a major obstacle to cancer therapy [23, 24]. Research on antisense lncRNAs has indicated their potential in therapeutic approaches; therefore, it necessary to summarize the roles and molecular mechanisms of antisense lncRNAs in the process of cancer development and progression. In this review, we present an overview of the main regulatory functions of antisense lncRNAs in different types of cancer types, as well as their potential clinical applications.

## Mechanisms of antisense lncRNA activity

Nuclear antisense lncRNAs contribute to the regulation of a large number of genes by either changing the condition of DNA via histone modifications and DNA modifications or recruiting specific factors to the DNA at transcriptional level. Cytoplasmic antisense lncRNAs [25], which are more abundant than nuclear ones, function as regulators of mRNA stability and translation. They can also sponge microRNAs, acting as competing-endogenous RNAs (ceRNAs). Furthermore, cytoplasmic antisense lncRNAs can bind to proteins to alter their half-life.

## Epigenetic regulations

**Fig. 2:**
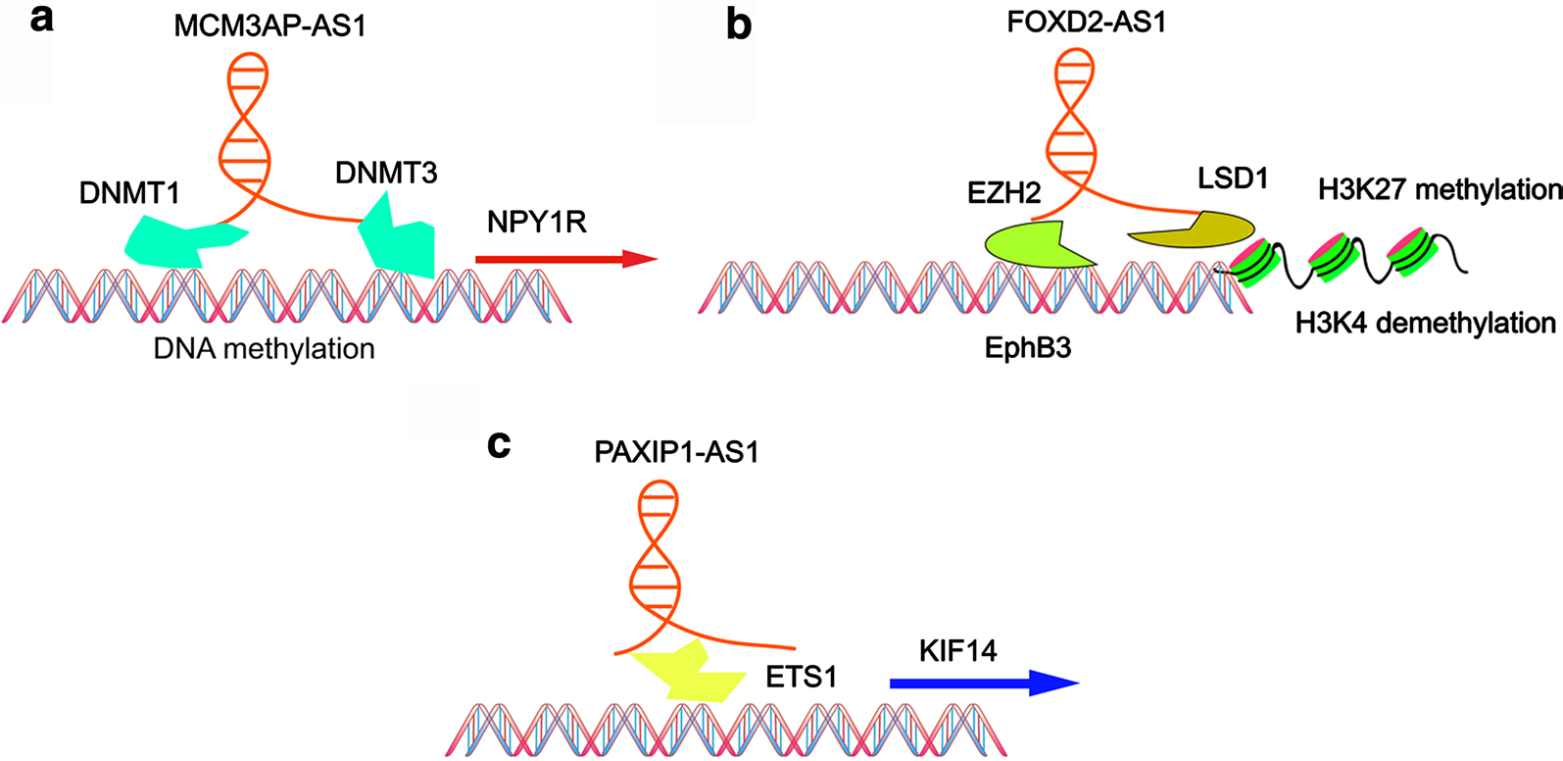
In the nucleus, antisense lncRNAs exert their roles through DNA methylation, histone modification and transcriptional gene regulation.** a** MCM3AP-AS1 recruits DNMT1/DNMT3 to the promoter of NPY1R, resulting DNA methylation.** b** FOXD2-AS1 interacts with EZH2 and LSD1 and recruits them to the promoter region of EphB3, respectively guiding H3K27 methylation and H3K4 demethylation.** c** PAXIP1-AS1 recruits transcription factor ETS1 to the promoter of KIF14 and further affects its transcription

Epigenetics is normally defined as heritable changes in gene expression without changes to the DNA sequence. Emerging research shows that antisense lncRNAs exert their role on gene expression through epigenetic modulations, such as DNA methylation and histone modifications. DNA methylation is an epigenetic process of regulating gene expression, and changes in DNA methylation patterns are very important for cancer development [26, 27]. Hypermethylation and hypomethylation of DNA both regulate the expression of oncogenes or tumor suppressors. There is a plethora of evidence linking antisense lncRNAs to the regulation of DNA methylation (Fig. 2a) [28]. For instance, Wu et al. demonstrated that antisense LncRNA *DLX6-AS1* is upregulated in liver cancer stem cells (LCSCs) and HCC, in which it functions as a oncogene and promotes the proliferation of LCSCs. Mechanistic studies indicated that downregulation of *DLX6-AS1* contributes to a reduction in *CADM1* promoter methylation via suppression of DNA methyltransferase 1 (DNMT1), DNMT3a, and DNMT3b, thus elevating *CADM1* expression in LCSCs and further inactivating the CADM1-dependent STAT3 signaling pathway [29]. Similarly, in prostate cancer (PCa), highly expressed *MCM3AP-AS1* facilitates cancer cell progression by recruiting DNMT1/DNMT3 to the *NPY1R* promoter, which downregulates *NPY1R* expression and activates the MAPK pathway [30]. Furthermore, *AFAP1-AS1* positively regulates the expression of the AFAP1 protein by negatively regulating CpG island methylation of the *AFAP1* promoter in lung cancer [31]. Overexpression of *ADAMTS9-AS2* results in the suppression of esophageal cancer development by inducing *CDH3* promoter methylation [32].

In addition to DNA methylation, chromatin structure and thus gene expression can be influenced by histone modifications [33]. Histone modifications are catalyzed by numerous histone-modifying enzymes, such as histone methyltransferases and histone acetyltransferases (Fig. 2b) [34]. In NSCLC, a high level of *AFAP1-AS1* expression correlates with poor clinical outcomes. Mechanistically, *AFAP1-AS1* interacts with EZH2, one type of histone methyltransferases, and recruits it to the promoter regions of *P21*, thus suppressing *P21* expression at epigenetic level [35]. In PCa, *ZEB1-AS1* interacts with the histone methyltransferase MLL1, a major methyltransferase responsible for the H3K4 modification. In this way, ZEB1-AS1 induces the H3K4me3 histone modification in the *ZEB1* promoter region, which activates the expression of *ZEB1* [36]. Besides recruiting methyltransferases, antisense lncRNAs can also recruit acetyltransferases. For example, in endometrial cancer, *DLX6-AS1* achieves its stimulative function by increasing *DLX6* expression via recruiting P300, a protein that can lead to histone acetylation in the *DLX6* promoter region [37]. Likewise, *AGAP2-AS1* promotes cell growth and inhibits apoptosis in breast cancer (BC) by inducing the histone acetylation in the *MYD88* promoter region [38]. In addition, a few antisense lncRNAs have been associated with other histone modifications. In gastric cancer (GC), upregulation of *FOXD2-AS1* promotes carcinogenesis by epigenetically silencing *EPHB3* via recruiting EZH2 and LSD1, leading to H3K27 methylation and H3K4 demethylation, respectively [39].

## Transcriptional modulation

At the transcriptional level, antisense lncRNAs regulate gene expression by recruiting transcription factors to the promoter of a specific gene [40]. Transcription factors play an important role in the process of transcription; they can bind with polymerase II and form a complex to further promote or repress gene expression (Fig. 2c) [40]. For instance, Xu et al. demonstrated that antisense lncRNA *PAXIP1-AS1* is highly expressed in glioma and correlated with poor prognosis. Functionally, upregulation of *PAXIP1-AS1* promotes migration, invasion, and angiogenesis of cancer cells. Mechanistic investigations indicated that *PAXIP1-AS1* recruits the transcription factor ETS1 to the promoter region of *KIF14* and further upregulates its expression [41]. Similarly, *TMPO-AS1* is upregulated and exerts its oncogenic roles in ovarian cancer (OC). Mechanistically, *TMPO-AS1* interacts with E2F6, a transcription factor that binds to the promoter region of *LCN2*, thus promoting *LCN2* transcription [42]. Another antisense lncRNA that exerts its function at the transcriptional level is *HOXB-AS1*. In glioblastoma (GBM), increased expression of *HOXB-AS1* promotes proliferation and induces apoptosis by recruiting transcription factor ILF3 to the promoter regions of *HOBX2* and *HOBX3* [43].

## Antisense LncRNAs acting as ceRNAs

**Fig. 3:**
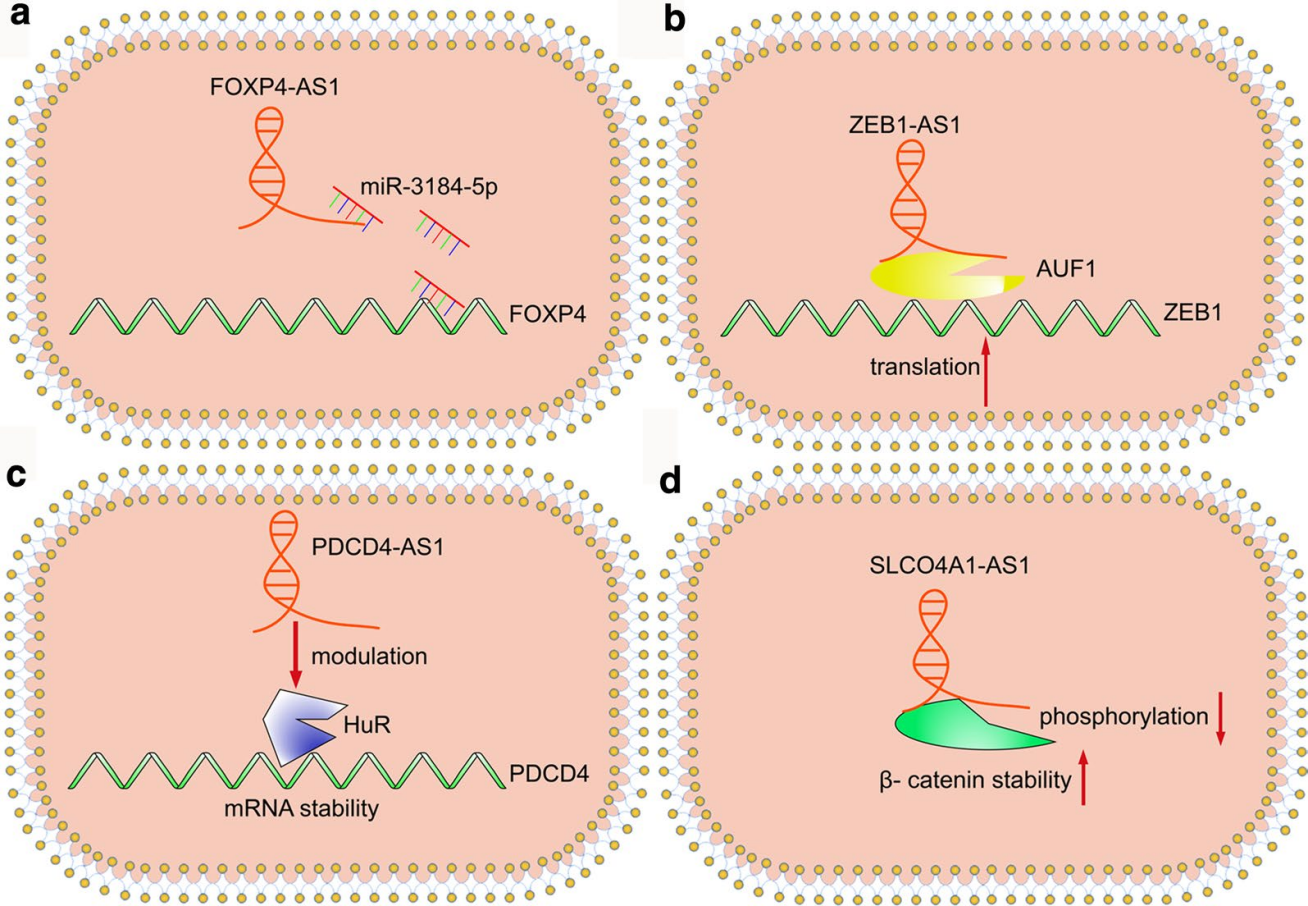
In the cytoplasm, antisense lncRNAs regulate cancer progression through post-trancriptional modulations and translational reprogramming.** a** the more miR-3184-5p molecules bind to FOXP4-AS1, the less miR-3184-5p molecules interact with FOXP4 mRNA, by this way, FOXP4-AS1 promotes tthe expression of FOXP4.** b** ZEB1-AS1 recruits AUF1 to ZEB1 mRNA and activates its translation without changing mRNAs level.** c** PDCD4-AS1 affects PDCD4 mRNA stability by regulating RNA-binding protein HuR binding to mRNA.** d** SLCO4A1-AS1 interacts with β-catenin and inhibites its phosphorylation

At the post-transcriptional level, numerous antisense lncRNAs serve as regulators of cancers by acting as ceRNAs. ceRNAs are the targets of microRNAs (miRNAs), and interact with miRNAs to further modulate the expression of the specific mRNA targeted by the miRNA (Fig. 3a) [44]. Through this miRNA-mediated method, antisense lncRNAs can affect cancer development.

In PCa cells, antisense lncRNA *FOXP4-AS1* and its corresponding coding transcript *FOXP4* are highly expressed. Upregulation of *FOXP4-AS1* correlates with poor prognosis and promotes cancer cell proliferation. Interestingly, *FOXP4-AS1* has the binding site for the *FOXP4*-targeting miRNA, miR-3184-5p. *FOXP4-AS1* competes with *FOXP4* for binding with miR-3184-5p. As a result, it positively regulates the FOXP4 protein level [45]. *ZEB1-AS1* tumor-promoter functions have been confirmed in many types of cancer, and there are two studies indicating that *ZEB1-AS1* promotes proliferation and migration of colorectal cancer (CRC) cells by acting as a ceRNA; however, the two targeted-miRNAs are different. Lv et al. found that there is an inverse correlation between *ZEB1-AS1* and miR-181a-5p levels in CRC cells. Further research demonstrated that *ZEB1-AS1* may function as a molecular sponge for miR-181a-5p [46]. By contrast, miR-101 is reported to function as tumor suppressor by targeting *ZEB1* in many types of cancer; therefore, Xiong et al. focused on the mechanism of the miR-101/*ZEB1* axis in CRC. Consistently, *ZEB1-AS1* knockdown, miR-101 overexpression, and ZEB1 depletion suppressed the proliferation and migration of CRC cells. *ZEB1-AS1* functions as a ceRNA for miR-101 and abrogated the silencing of *ZEB1* caused by miR-101 [47]. *ZEB1-AS1* participates in tumorigenesis and progression in various cancer types, and it is likely that more miRNA targets will be found in the future. Moreover, miR-1253 is the target of *FOXC2-AS1* in PCa [48] and *TPT1-AS1* acts as a sponge for miR-324-5p in cervical cancer (CC) [49]. It is becoming clear that many antisense LncRNAs exert their roles in cancer via this ceRNA mechanism, thus future works in this area might lead to the development of promising therapeutics.

## Translational control by antisense lncRNAs

Regulation of gene expression is not limited to epigenetic and transcriptional regulatory networks, antisense LncRNAs can also regulate gene expression at the translational level. Protein synthesis is controlled by numerous tumor suppressors and oncogenes, making it easy to respond to environmental changes by regulating this process. Antisense lncRNAs are involved in regulating protein synthesis and degradation. Firstly, they can recruit the target of mRNAs so as to affect their translation (Fig. 3b). For instance, in bladder cancer (BCa) cells, Zhao et al. demonstrated that *ZEB1-AS1* expression is higher in comparison with that in corresponding normal tissues, which promotes BCa cells migration and invasion. Mechanistically, they found that *ZEB1-AS1* upregulates the expression of ZEB1 without increasing its mRNA level. Unexpectedly, it activates the translation of *ZEB1* mRNA by recruiting AUF1, which is able to bind to (A + U)-rich elements within 3ʹ-untranslated region (3ʹ-UTR) of target mRNA and promote translation without affecting the mRNA level [50].

Secondly, antisense lncRNAs affect the stability of mRNA by regulating the association of RNA-binding proteins with mRNA (Fig. 3c). PDCD4 is a tumor suppressor in BC, and the expression level of PDCD4 correlates positively with the level of antisense lncRNA *PDCD4-AS1*. Mechanistically, overexpression of *PDCD4-AS1* increases the level of *PDCD4* mRNA. To rule out the possibility that *PDCD4-AS1* regulates *PDCD4* expression at the epigenetic or transcriptional level, researchers quantified the levels of *PDCD4* pre-mRNA, which showed that there was no significant change in the level of *PDCD4* pre-mRNA in *PDCD4-AS1* deleted cells compared with that in the control group. This indicated that *PDCD4-AS1* increases the level of *PDCD4* mRNA by improving its stability. Additional investigations demonstrated that *PDCD4-AS1* promotes *PDCD4* mRNA stability by negatively modulating HuR [51].

Likewise, in BC, *CERS6-AS1* functions as a cancer promoter by binding to IGF2BP3, which increases the stability of *CERS6* mRNA [52]. *HOXB-AS1* facilitates cell growth in multiple myeloma by binding to ELAVL1, thus promoting *FUT4* mRNA stability [53].

Finally, antisense lncRNAs can affect the level of certain proteins by modulating the process of protein degradation by prolonging or shortening protein half-life (Fig. 3d). For example, *SLCO4A1-AS1* was confirmed as a tumor-promoter antisense lncRNA in CRC, in which the level of *SLCO4A1-AS1* correlated positively with the level of β-catenin. Further investigations indicated that *SLCO4A1-AS1* can interact with β-catenin and increase its stability by inhibiting its phosphorylation [54]. *ZFPM2-AS1* expression is higher in GC cells than in normal gastric tissue. By binding to and stabilizing macrophage migration inhibitory factor (MIF), the suppressor of p53 stability, increased levels of *ZFPM2-AS1* promote proliferation and suppresses apoptosis of cancer cells [55]. Likewise, *FEZF1-AS1* promotes CRC cell proliferation and metastasis through activation of the STAT3 signaling pathway by increasing the stability of the pyruvate kinase 2 (PKM2) [56].

## The difference between lncRNAs and antisense lncRNAs

Nucleotide sequence complementarity allows antisense lncRNA to have special effects on their sense gene, thus they are more likely to regulate the expression of their corresponding protein-coding gene, which contrasts with other types of lncRNAs. A good way to find out how antisense lncRNAs affect the growth of cancer cells is to detect the expression of its sense gene.

In prostate cancer, a correlation between the level *ZEB1-AS*1 and ZEB1 was demonstrated. *ZEB1-AS1* recruits histone methyltransferase MLL1 to the promoter region of *ZEB1*, thus inducing the H3K4me3 modification, and activating *ZEB1* transcription [36]. Similarly, *ZNF667-AS1* and its sense gene, *ZNF667*, are downregulated in esophageal squamous cell carcinoma. *ZNF667-AS1* affects the expression of *ZNF667* via promoter CpG site methylation by recruiting TET1, which can hydrolyze 5′-methylcytosine (5′-mc) to 5′-hydroxymethylcytosine (5′-hmc) [57]. Many histone-modifying enzymes cannot exert their role independently because they lack specific DNA-binding domains, thus a large portion of antisense lncRNAs bind chromatin-modifying enzymes and recruit them to their sense gene [15]. In addition, at the translational level, antisense lncRNAs can directly bind with their sense mRNA and form an RNA duplex, which affects the stability of these targeted mRNAs. For instance, in skin cutaneous carcinoma, *TTN-AS1* directly regulates *TTN* expression by forming a RNA duplex with *TTN* mRNA [58]. In addition, the overlapping region of *UPK1A-AS1* increases the stability of *UPK1A* mRNA by forming a duplex in lung cancer cells [59].

## Antisense lncRNAs in tumorigenesis and progression

**Fig. 4:**
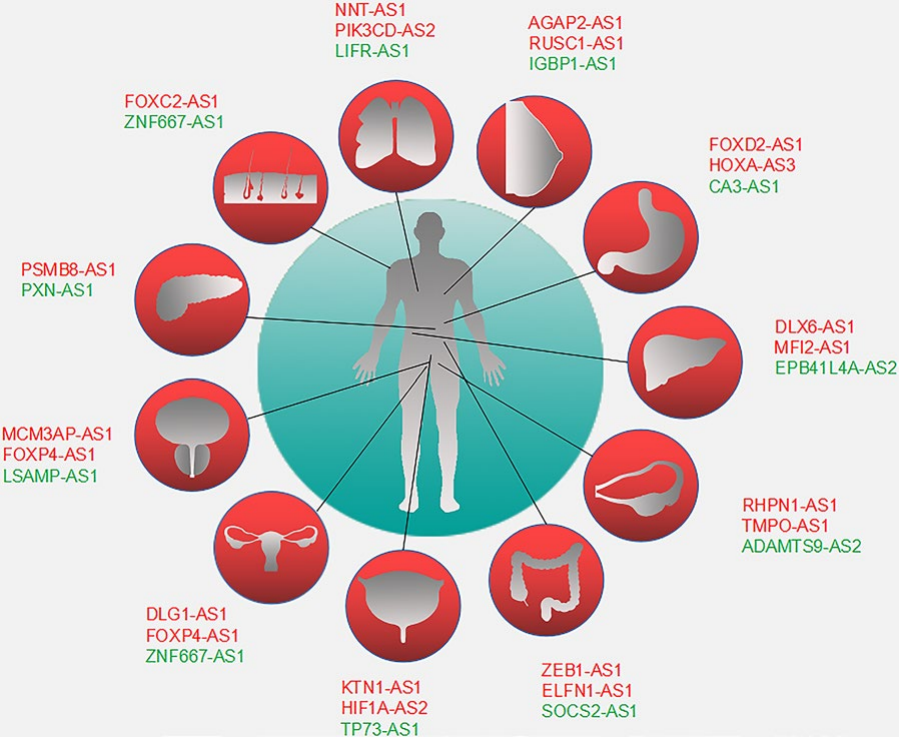
Antisense lncRNAs involve in many types of cancer. Antisense lncRNAs function as oncogene (red) and tumor-suppressor (green) in different cancer types. Clockwise from top left: lung cancer, breast cancer, gastric cancer, liver cancer, ovarian cancer, colorectal cancer, bladder cancer, cervical cancer, prostate cancer, pancreatic cancer, melanoma

Antisense lncRNAs have a crucial effect in the process of tumor development and progression in various cancer types, either acting as oncogenes or tumor suppressors. Interestingly, the function of some antisense lncRNAs depends on the type of cancer, functioning as oncogenic factor in some cancers, while acting a tumor suppressor in other cancer types [60]. In this section, we provide relevant examples of well-established antisense lncRNAs having oncogenic, tumor suppressive, or dual properties (Fig. 4) [61].

## Antisense lncRNAs function as oncogenes

In this part, we discuss how antisense lncRNAs promote cancer cell proliferation and migration. Among the numerous oncogenic antisense lncRNAs, we focus on *KTN1-AS1* and *FOXP4-AS1*, whose oncogenic functions have been confirmed in different cancer types. *KTN1-AS1* is reported to be highly expressed in six types of cancer. In NSCLC, STAT1-induced upregulation of *KTN1-AS1* facilitates cancer cell progression via the miR-23b/*DEPDC1* axis [62]. In BCa, *KTN1-AS1* knockdown inhibited the proliferation and invasion of cancer cells. Mechanistically, *KTN1-AS1* recruits EP300, a histone acetyltransferase, which enriched H3K27Ac in the *KTN1* promoter region, thus activating the expression of *KTN1* [63]. Furthermore, *KTN1-AS1* increases the viability and invasive ability of glioma cells in vitro and in vivo through the *KTN1-AS1*/miR-505-3p pathway and promotes tumor growth of HCC via the miR-23c/*ERBB2IP* axis [64, 65].

Antisense lncRNAs forkhead box P4 antisense RNA 1, known as *FOXP4-AS1*, is significantly overexpressed in approximately 10 types of human cancers. For example, in mantle cell lymphoma, *FOXP4-AS1* accelerates the progression of cancer by sponging miR-136-5p to further regulate the downstream target of miR-136-5p, *NACC1* [66]. Interestingly, *FOXP4-AS1* promotes CC progression by binding with miR-136-5p, the same target microRNA as that in mantle cell lymphoma, indicating the generality of cancer development between different cancer types. Subsequent studies demonstrated that the target of miR-136-5p is not *NACC1* but *CBX4* in CC [67]. Likewise, in nasopharyngeal carcinoma, *FOXP4-AS1* promotes cancer cell proliferation and inhibits apoptosis via the miR-423-5p/*STMN1* axis [68]. Moreover, through the miR-3184-5p/*FOXP4* axis, *FOXP4-AS1* promotes the proliferation of esophageal squamous cell carcinoma cells [69].

## Antisense lncRNAs act as tumor suppressors

The expression of some antisense lncRNAs is downregulated in cancer cells, because they inhibit cancer cell proliferation, migration, and invasion. Here, we discuss three antisense lncRNAs, HAND2-AS1, FGF13-AS1, and FGF14-AS2, which exert tumor suppressive roles during the onset and progression of cancer.

Antisense lncRNA *HAND2-AS1*, transcribed from the opposite strand of *HAND2* (encoding heart and neural crest derivatives expressed 2) on chromosome 4q33-34, was first reported to be downregulated in endometrioid endometrial carcinoma (EEC). Its anti-tumorigenic effect is mediated by downregulating NMU, an oncogenic protein in EEC [70]. In GC cells, *HAND2-AS1* expression is decreased; however, overexpression of *HAND2-AS1* is capable of inhibiting GC cell proliferation and promoting their apoptosis by functioning as a ceRNA that binds with miR-590-3p [71]. Another study demonstrated that *HAND2-AS1* can also exert its tumor suppressive role through the miR-769-5p/*TCEAL7* axis in GC [72]. In HCC, *HAND2-AS1* overexpression reduces the viability and proliferation of cancer cells by sponging miR-300 [73]. Furthermore, in NSCLC cells, *HAND2-AS1* represses the proliferation of cancer cells by targeting the PI3K/Akt pathway [74].

The role of *FGF13-AS1* in tumors has only been reported in BC, in which it suppresses BC cell proliferation, migration, and invasion by impairing glycolysis and stemness properties. Mechanistically, *FGF13-AS1* shortens the half-life of *MYC* mRNA by interacting with the RNA-binding protein IGF2BPs and further interrupting the interaction between IGF2BPs and *MYC* mRNA, resulting in the suppressed expression of c-Myc. Simultaneously, downregulated c-Myc transcriptionally inhibits *FGF13-AS1*, forming a feedback loop [75].

*FGF14-AS2* was first identified as a tumor suppressor in BC. Compared with that in adjacent normal tissue, *FGF14-AS2* is significantly downregulated in BC tissues [76]. As reported by Jin and coworkers, *FGF14-AS2* activates the expression of *FGF14* at the post-transcriptional level by functioning as a ceRNA of miR-370-3p in BC [77]. Moreover, it sponges miR-1288-3p, which indirectly controls Ras/ERK signaling, causing inhibition of CRC proliferation [78].

## Antisense LncRNAs with dual activity

A few antisense lncRNAs have been reported to play opposite roles in different types of cancer. These inconsistent functions could be partly explained by the wide genetic and phenotypic heterogeneity of tumors, and the different experimental methods and samples used. Herein, three confirmed examples of antisense lncRNAs with divergent roles in tumors are discussed.

A tumor-stimulative role of *TP73-AS1* has been reported in various types of solid tumor, including lung, breast, gastric, and hepatic carcinomas. Mechanistically, it exerts its activity on tumor proliferation mostly by functioning as a ceRNA. In lung cancer, *TP73-AS1* knockdown inhibited the growth and metastasis of cancer cells through the miR-27b-3p/*LAPTM4B* axis [79]. In BC, *TP73-AS1* sponges miR-200a, indirectly activating the expression of *ZEB1* and promoting cell proliferation [80]. Similarly, in HCC, overexpression of *TP73-AS1* competes with *HMGB1* for miR-200a binding, causing the upregulation of HMGB1, a critical regulator of cell death and survival [81]. However, *TP73-AS1* was confirmed to be downregulated in acute myeloid leukemia (AML), which affects the cell proliferation of AML through the miR-21/*PTEN* axis [82]. A tumor-suppressive role of *TP73-AS1* has been also reported in BCa, patients with low *TP73-AS1* expression have shorter disease-free survival than patients with high *TP73-AS1* expression. Further investigations indicated that *TP73-AS1* functions as a tumor suppressor via its role in epithelial-mesenchymal transition (EMT) [83].

*ADAMTS9-AS2* has been reported to have either an oncogenic or tumor suppressive function. In GC, *ADAMTS9-AS2* acts as a tumor suppressor via its ability to activate NLRP3-mediated pyroptotic cell death through sponging miR-223-3p [84]. In OC, its downregulation correlated with lymph-node metastasis and poor overall survival. *ADAMTS9-AS2* inhibits OC progression by regulating the miR-182-5p/*FOXF2* axis [85]. However, high *ADAMTS9-AS2* expression was observed in tongue squamous cell carcinoma (TSCC), in which it shows an explicitly oncogenic role in tumorigenesis by competing wit̄h miR-600 [86].

Except in myeloid malignancy, *LEF1-AS1* has been identified as an oncogene in all cancer types reported to date. In NSCLC, *LEF1-AS1* promotes cancer cell proliferation and inhibits their apoptosis by regulating the miR-221/*PTEN* pathway [87]. Similarly, it functions as a oncogenic factor through the miR-30-5p/*SOX9* axis in colon cancer and boosts the proliferation, migration, and invasion of osteosarcoma by increasing the mRNA stability of *LEF1* [88, 89]. Nevertheless, downregulation of *LEF1-AS1* correlates positively with tumor progression in patients with myelodysplastic syndrome and acute myeloid malignancy, indicating a tumor suppressive role in myeloid malignancy [90].

As summarized in Table 1, we have distilled the conclusions from many studies and present the mechanisms by which antisense lncRNAs affect tumor development and progression.

**Table 1:** Antisense LncRNAs act as oncogenes or tumor suppressors in various cancer types

Antisense LncRNA	Cancer type	Function	Mechanism	Refs
MFI2-AS1	Liver cancer	Oncogene	MFI2-AS1 functions as miR-134 sponge to Upregulate FOXM1 expression	[122]
EPB41L4A-AS2		Tumor suppressor	EPB41L4A-AS2 sponges miR-301a-5p and targets FOXL1	[123]
PIK3CD-AS2	Lung cancer	Oncogene	PIK3CD-AS2 suppresses p53 pathway via YBX1	[124]
LIFR-AS1		Tumor suppressor	LIFR-AS1 regulates miR-942-5p/ZNF471 axis	[125]
ZNFX1-AS1	Bladder cancer	Oncogene	ZNFX1-AS1 interacts with miR-193a-3p/Syndecan 1	[126]
MAGI2-AS3		Tumor suppressor	MAGI2-AS3 upregulates TNS1 by sponging miR-31-5p	[127]
RUSC1-AS1	Breast cancer	Oncogene	RUSC1-AS1 downregulates the expression of CDKN1A and KLF2	[128]
IGBP1-AS1		Tumor suppressor	IGBP1-AS1 modulates miR-24-1/ZIC3 axis	[129]
ELFN1-AS1	Colorectal cancer	Oncogene	ELFN1-AS1 acts as a sponge of miR-4644 to increase TRIM44 expression	[130]
SOCS2-AS1		Tumor suppressor	SOCS2-AS1 stabilizes SOCS2 and sponges miR-1264	[131]
CTBP1-AS2	Cervical cancer	Oncogene	CTBP1-AS2 upregulates ZNF217 through sponging miR-3163	[132]
ZNF667-AS1		Tumor suppressor	ZNF667-AS1 counteracts microRNA-93-3p-dependent PEG3 downregulation	[133]
HOXA-AS3	Gastric cancer	Oncogene	HOXA-AS3 activates NF-κB signaling through miR-29a-3p/LTβR axis	[134]
CA3-AS1		Tumor suppressor	CA3-AS1 sponges miR-93-5p and targets BTG3	[135]
VPS9D1-AS1	Prostate cancer	Oncogene	VPS9D1-AS1 sponges miR-4739 to upregulate MEF2D	[136]
LSAMP-AS1		Tumor suppressor	LSAMP-AS1 binds to microRNA-183-5p and upregulates the tumor suppressor DCN	[137]
RHPN1-AS1	Ovarian cancer	Oncogene	RHPN1-AS1 acts as a ceRNA against miR-596 and upregulating LETM1	[114]
ZNF667-AS1	Melanoma	Tumor suppressor	ZNF667-AS1 positively regulates MEGF10	[138]
FOXC2-AS1		Oncogene	FOXC2-AS1 downregulates p15 by recruiting EZH2	[139]
PSMB8-AS1	Pancreatic cancer	Oncogene	PSMB8-AS1 modulates miR-382-3p/STAT1/PD-L1 axis	[140]
PXN-AS1		Tumor suppressor	PXN-AS1 acts as a ceRNA of miR-3064 to upregulate PIP4K2B expression	[141]

## Antisense lncRNAs in chemoradioresistance

In addition to surgery, chemotherapy and radiation therapy are the two effective methods to improve the survival rate and prognosis of people with cancer. However, chemoradioresistance represents a major barrier to tumor therapy; therefore, it is necessary to determine the mechanism underlying a tumor chemoradioresistance.

Recently, increasing evidence indicates that the drug-resistant tumor phenotype is regulated by the expression of certain genes [91], and antisense lncRNAs are also reported to be involved in this process. Below, we discuss two antisense lncRNAs whose drug-resistance activities have been determined in some types of cancer, more examples are presented in Table 2.

**Table 2:** Antisense LncRNAs are related to drug resistance in cancer

Antisense LncRNA	Cancer type	Drug	Mechanism	Refs
HOXD-AS1	Cervical cancer	Cisplatin	HOXD-AS1 enhances chemoresistance of cisplatin-resistant cancer cells by modulating miR-130a-3p/ZEB1 axis	[142]
DLX6-AS1	Breast cancer		DLX6-AS1 promotes cisplatin resistance through miR-199b-5p/PXN signaling	[143]
NCK1-AS1	Osteosarcoma		NCK1-AS1 knockdown enhances Cisplatin sensitivity of cancer cells by regulating miR-137	[144]
SLC7A11-AS1	Pancreatic cancer	Gemcitabine	SLC7A11-AS1 promotes Gemcitabine-resistance by Blocking SCF β-TRCP-Mediated Degradation of NRF2	[145]
SBF2-AS1	Pancreatic cancer		SBF2-AS1 promotes the expression of TWF1 by binding with miR-142-3p to induce gemcitabine resistance	[146]
LOXL1-AS1	Prostate cancer	Doxorubicin	LOXL1-AS1/miR-let-7a-5p/EGFR-related pathway regulates the doxorubicin resistance	[147]
FOXC2-AS1	Osteosarcoma		FOXC2-AS1 promotes doxorubicin resistance by increasing the expression of FOXC2	[148]
AFAP1-AS1	Breast cancer	Trastuzumab	AFAP1-AS1 promotes trastuzumab resistance by binding with AUF1 and activating ERBB2 expression	[149]
SBF2-AS1	Glioblastoma	Temozolomide	SBF2-AS1 enhances chemoresistance to temozolomide by functioning as a ceRNA for miR-151a-3p	[150]
ADAMTS9-AS2	Glioblastoma		ADAMTS9-AS2 promotes Temozolomide Resistance via Upregulating the FUS/MDM2 Ubiquitination Axis	[151]
NR2F1-AS1	Liver cancer	Oxaliplatin	NR2F1-AS1 regulates oxaliplatin resistance by targeting ABCC1 via miR-363	[152]
DSCAM-AS1	Breast cancer	Tamoxifen	DSCAM-AS1 enhances Tamoxifen resistance by functioning as a sponge of miR-137	[153]
ADAMTS9-AS2	Breast cancer		ADAMTS9-AS2 enhances tamoxifen resistance by activating miR-130a-5p	[154]
AFAP1-AS1	Prostate cancer	Paclitaxel	AFAP1-AS1 modulates the sensitivity of paclitaxel via miR-195-5p/FKBP1A axis	[155]
DDX11-AS1	Esophageal cancer		DDX11-AS1 promotes resistance cancer cells to Paclitaxel by inhibiting TOP2A expression via TAF1	[156]

In esophageal squamous cell carcinoma, Liu et al. demonstrated that *FXOD2-AS1* overexpression promotes cisplatin resistance through the miR-195/*Akt/mTOR* axis [92]. In glioma, *FOXD2-AS1* functions as a prognostic factor and induces temozolomide resistance in a *O*(6)-methylguanine-DNA methyltransferase-dependent manner [93]. Meanwhile, *FOXD2-AS1* might also contribute to temozolomide resistance in glioma via the miR-98-5p/*CPEB4* axis [94]. By promoting *STAT3* transcriptional activity, *FOXD2-AS1* enhances chemotherapy resistance of laryngeal squamous cell carcinoma [95]. Furthermore, *FOXD2-AS*1 binds with miR-143, leading to gemcitabine-resistance in BCa [96].

*OIP5-AS1* is more likely to function as a ceRNA when playing its role in drug resistance. In osteosarcoma, *OIP5-AS1* mediates resistance to doxorubicin by regulating the miR-137-3p/*PTN* axis [97]. In addition, *OIP5-AS1* either modulates the miR-377-3p/FOSL2 signaling pathway or induces the LPAATβ/PI3K/AKT/mTOR signaling pathway by sponging miR-340-5p, thus regulating cisplatin sensitivity [98, 99]. Similarly, in colon cancer, *OIP5-AS1* regulates drug-resistance to oxaliplatin by sponging miR-137 [100].

Increasing numbers of studies have focused on the mechanisms by which antisense lncRNAs affect drug sensitivity to cancer, covering various types of chemotherapeutic drugs in different cancers; therefore, providing a new direction to solve this problem.

Similarly, radiation therapy is a very common treatment for many types of cancer, either alone or in combination with other therapeutic methods. The effect greatly depends on the radiosensitivity of the cancer cells. Patients require a higher dose of irradiation when the tumor is resistant to radiation therapy, resulting in more damage to normal tissues. Some studies reported that dysregulation of antisense lncRNAs might be involved in this process. The expression level of certain antisense lncRNAs is different between radioresistant and radiosensitive tumors, indicating that modulation of their expression could improve the radiosensitivity of tumors. In OC, the marked upregulation of *FAM83H-AS1* contributes to radioresistance by increasing the stability of HuR, an RNA binding protein that had been reported to regulate radioresistance in multiple cancers [101]. In NSCLC, upregulated *SBF2-AS1* reduces the radiosensitivity and apoptosis of cancer cells via regulating the miR-302a/*MBNL3* axis [102]. *PTPRG-AS1* promotes radioresistance in two cancer types: in nasopharyngeal carcinoma (NPC), *PTPRG-AS1* reduces sensitivity to radiotherapy through the miR-194-3p/*PRC1* regulatory axis [103]; whereas, under X-ray irradiation, overexpression of *PTPRG-AS1* could promote the viability and enhance the radioresistance of NSCLS by modulating the miR-200c-3p/*TCF4* axis [104]. In a similar role, *TTN-AS1* sponges miR-134-5p to regulate the radiosensitivity of human large intestine cancer cells [105]. It is evident that the modulation of antisense lncRNA expression can be used to improve the radiosensitivity of tumors, providing a new method to solve the problem of radioresistance in cancer.

## Antisense LncRNA databases

Online databases are good tools to understand dysregulated lncRNA, simultaneously, these databases can also be used to understand antisense LncRNA. Among the many databases containing information related to lncRNAs, we would like to introduce three particularly useful databases.

### NONCODE

NONCODE (http://www.noncode.org/) is an integrated knowledge database dedicated to collecting information regarding noncoding RNA. Recently, it was updated to v6.0. Almost all types of ncRNA (excluding tRNAs and rRNAs) are covered, not only providing basic information, such as the location, sequence, and source, but also advanced information, such as the expression profile and conservation information. In the current version, there are 39 species (16 animals and 23 plants), representing an increase of 22 compared with v5.0. NONCODE has collected a total of 173,112 human lncRNAs, and v6.0 contains updated human lncRNA-cancer relationships, which will help us to explore the roles of lncRNAs in cancer [106].

### LncRNADisease

LncRNADisease v2.0 (http://www.rnanut.net/lncrnadisease/) focuses on the relationship between diseases and lncRNAs, collecting experimentally supported lncRNA-disease associations. In comparison with the previous version, LncRNADisease v2.0 has an over 40-fold increase in lncRNA-disease associations. There is a confidence score system to evaluate the reliability of the relationship between a disease and an lncRNA. A score close to 1 represents a strong association. Besides, to further explore the network of lncRNAs with mRNAs and miRNAs, LncRNADisease v2.0 covers 12,207 lncRNA–mRNA and 2368 miRNA–lncRNA regulatory relationships, and an lncRNA–miRNA–mRNA network has also been constructed [107].

### LNCipedia

LNCipedia 5 (https://lncipedia.org) contains a total of 56,946 lncRNA genes and 127,802 lncRNA transcripts. Compared with other databases, LNCipedia has an advantage: in the current version, 6% of the genes and 23% of the transcripts are annotated with an official gene symbol, making it more convenient to study lncRNAs. Moreover, in the advanced search, we can choose the class as antisense, making it easier to find dysregulated antisense lncRNAs [108].

## Potential applications

Antisense lncRNAs are highly tissue-specific drivers of cancer phenotypes and are identified as crucial regulators associated with tumorigenesis and suppression, showing great potential, not only as biomarkers, but also as therapeutic targets for cancer treatment. Antisense lncRNAs have been found to be involved in all steps of cancer development and progression. First, antisense lncRNAs regulate the proliferation, migration, invasion, and apoptosis of cancer cells, which means they can function as diagnostic biomarkers. Second, the expression levels of some antisense lncRNAs are associated with tumor size and TNM stage; therefore, they could be used to evaluate tumorigenesis and cancer progression. Furthermore, the levels of some antisense lncRNAs correlate with certain prognostic markers, indicating their ability to predict cancer prognosis. For example, in HCC, *SOX21-AS1* is a highly expressed antisense LncRNA that acts as an oncogene in cancer cell proliferation and cell cycle progress. Further investigations indicated that the expression level of *SOX21-AS1* correlated with tumor size, Edmondson Grade, vascular invasion, and cirrhosis. Kaplan–Meier analysis showed that patients with HCC with high levels of *SOX21-AS1* expression had a shorter survival time compared with those with low expression of *SOX21-AS1*. These results demonstrated that *SOX21-AS1* is a potential biomarker for HCC [109]. Likewise, downregulated *ZNF385D-AS2* is predictive of poor prognosis of patients with liver cancer [110]. In addition, *TMPO-AS1* and *FOXC2-AS1* are implicated as biomarkers for PCa [48, 111].

Antisense lncRNAs are differentially expressed in different cancer types and their expression levels are related to tumorigenesis and aggressiveness, making them potential targets for cancer treatment. Targeting antisense lncRNAs and modulating their expression could affect many biological processes of cancer cells. In NSCLC, *NNT-AS1* expression is upregulated in cancer cell lines; therefore, to explore the roles of *NNT-AS1* in NSCLC, cancer cells were transfected with a small interfering RNA si-(NNT-AS1) and a negative control, si-NC. The results indicated that the migration ability of cancer cells in the si-NNT-AS1 group was suppressed compared with that in the si-NC group. In addition, the invasion ability of cancer cells transfected with si-NNT-AS1 was suppressed compared with that in the si-NC group [112]. Similarly, in BC, *HIF1A-AS2* is upregulated, and researchers transfected a short hairpin RNA (shRNA), sh-HIF1A-AS2, into cancer cells to reduce the level of *HIF1A-AS2*. The results showed that the proliferation capacity of the cancer cells transfected with sh-HIF1A-AS2 was significantly reduced, as were the levels of proliferation marker proteins. Through different ways of targeting antisense lncRNAs and reducing their expression, the growth of cancer cells was suppressed [113]. Meanwhile, this effect also exists in vivo, which further confirms the therapeutic value of targeting antisense lncRNAs. In epithelial ovarian cancer, highly-expressed *RHPN1-AS1* was suppressed using an shRNA. Cells were injected into mice and grown for 6 weeks. The results showed that knockdown of *RHPN1-AS1* significantly reduced the growth of epithelial ovarian cancer in the xenograft tumor model [114]. In another study, researchers treated cancer cells with lentiviral CRISPR/Cas9 to stably knockout *DSCAM-AS1*, which inhibited the growth of MCF7 xenograft tumors when compared with the negative control group [115]. In addition, as mentioned above, antisense lncRNAs also have great potential to solve the problems of cancer cell resistance to chemotherapy and radiotherapy.

## Challenges to the application of antisense lncRNAs

There are thousands of articles reporting on the relationships between antisense lncRNAs and cancer, providing researchers with a lot of data. These data are the basis for future study; however, similar research sometimes produces conflicting conclusions. For instance, in ovarian cancer, Miao et al. demonstrated that *TTN-AS1* expression is decreased in cancer tissues and cells. TTN-AS1 inhibits the cell growth of OC through the miR-15b-5p/*FBXW7* axis, as demonstrated in several OC cell lines [116]. However, Liu et al. indicated that a high level of *TTN-AS1* is found in OC tissues and cell lines, in which *TTN-AS1* promotes the progression of OC by modulating the miR-139-5p/*ROCK2* axis, and their samples were mainly obtained from patients with OC [117]. Similarly, three studies on the relationship between *LIFR-AS1* and GC reported contradictory results. Their cancer tissues were collected from patients with GC from different areas [118–120]. Ignoring the experimental errors, the different experimental samples might have resulted in the presence of different cancer subtypes, which might have partly contributed to the generation of conflicting results. Therefore, it might be necessary to investigate how different cancer subtypes affect the role of antisense lncRNAs on cancer. By contrast, although we have revealed the mechanisms by which antisense lncRNAs affect the process of cancer development, the present method to detect the expression level of antisense lncRNA is not very useful; therefore, it might be better to identify antisense lncRNA candidates whose expression is easy to monitor. There is still a long way to go to apply these results to clinical practice. Lastly, some antisense lncRNAs have been studied; however, the functions of the majority of these transcripts remain to be determined [121]. Further investigations of antisense lncRNAs will provide more possibilities for cancer diagnostics and therapy.

## Conclusion

Growing evidence demonstrates that many antisense lncRNAs are dysregulated in cancer cells. Antisense lncRNAs play a crucial role in tumor onset, progression, chemotherapy responses, and radiotherapy sensitivity by regulating gene and protein expression at epigenetic, transcriptional, post-transcriptional, and translational levels. The close relationship between antisense lncRNAs and cancers mean that antisense lncRNAs have great potential as biomarkers to diagnose cancer, predict prognosis, and as targets for tumor treatment. However, we cannot ignore the difficulty of applying antisense lncRNA-based therapeutic approaches in the clinic. Additional research will provide more hope of finding a cure for cancer.

## Data Availability

Not applicable.

## Abbreviations

antisense lncRNA: Antisense long non-coding RNA
ncRNA: Non-coding RNA
lncRNA: long non-coding RNA
CDKN2B-AS1: Cyclin-dependent kinase inhibitor 2B antisense RNA 1
HCC: Hepatocellular carcinoma
NSCLC: Non-small cell lung cancer
NNT-AS1: Nicotinamide nucleotide transhydrogenase-antisense 1
TMPO-AS1: Thymopoietin antisense transcript 1
DLG1-AS1: DLG1 antisense RNA 1
PC: Prostate cancer
CC: Cervical cancer
DLX6-AS1: Distal-less homeobox 6 antisense RNA 1
LCSC: Liver cancer stem cell
CADM1: Cell adhesion molecule 1
DNMT: Methyltransferase
STAT3: Signal transducer and activator of transcription 3
AFAP1-AS1: Actin filament associated protein 1 antisense RNA 1
ADAMTS9-AS2: ADAM metallopeptidase with thrombospondin type 1 motif, 9 antisense RNA 2
CDH3: Cadherin 3
EZH2: Enhancer of Zeste Homolog 2
ZEB1-AS1: Zinc finger E-box binding homeobox1-antisense RNA 1
MLL1: Mixed lineage leukaemia protein-1
H3K4: Histone 3 lysine 4
AGAP2-AS1: Arf GAP with GTP-binding protein-like domain, Ankyrin repeat and PH domain 2 antisense RNA 1
BC: Breast cancer
MyD88: Myeloid differentiation primary response protein 88
GC: Gastric cancer
FOXD2-AS1: FOXD2 adjacent opposite strand RNA 1
EphB3: Ephrin type-B receptor 3
LSD1: Lysine-specific demethylase 1
PAXIP1-AS1: PAX-interacting protein 1-antisense RNA1
ETS1: ETS proto-oncogene 1
KIF14: Kinesin family member 14
OC: Ovarian cancer
E2F6: E2F transcription factor 6
LCN2: Lipocalin-2
HOXB-AS1: Homeobox B cluster antisense RNA 1
GBM: Glioblastoma
ILF3: Interleukin enhancer-binding factor 3
ceRNA: Competing endogenous RNA
miRNA: Micro RNA
FOXP4-AS1: Forkhead box P4 antisense RNA 1
CRC: Colorectal cancer
FOXC2-AS1: Forkhead Box C2 antisense RNA 1
TPT1-AS1: Tumor protein translationally controlled 1 antisense RNA 1
BCa: Bladder cancer
AUF1: AU-rich element RNA-binding factor 1
PDCD4-AS1: Programmed cell death 4 antisense RNA 1
MACC1-AS1: Metastasis associated in colon cancer-1 antisense RNA 1
SLCO4A1-AS1: SLCO4A1 antisense RNA 1
ZFPM2-AS1: Zinc finger protein multitype 2 antisense RNA 1
MIF: Migration inhibitory factor
FEZF1-AS1: FEZ finger zinc 1 antisense 1
PKM2: Pyruvate kinase 2
KTN1-AS1: Kinectin 1-Antisense RNA 1
DEPDC1: DEP domain containing 1
ERBB2IP: Erbb2 interacting protein
NACC1: Nucleus accumbens-associated protein 1
CBX4: Chromobox homolog 4
STMN1: Stathmin 1
HAND2-AS1: Heart and neural crest derivatives expressed 2-antisense RNA 1
KCNT2: Potassium sodium-activated channel subfamily T member 2
TCEAL7: Transcription Elongation Factor A-like 7
FGF13-AS1: Fibroblast growth factor 13 antisense RNA 1
IGF2BPs: Insulin-like growth factor-2 mRNA-binding proteins
FGF14-AS2: Fibroblast Growth Factor 14 antisense RNA 2
TP73-AS1: Tumour protein P73 antisense RNA 1
LAPTM4B: Lysosomal-associated transmembrane protein 4B
AML: Acute myeloid leukemia
PTEN: Phosphatase and Tensin Homolog deleted on Chromosome 10
EMT: Epithelial–mesenchymal transition
NLRP3: NOD-, LRR- and pyrin domain-containing 3
OC: Ovarian cancer
FOXF2: Forkhead box F2
TSCC: Tongue squamous cell carcinoma
LEF1-AS1: Lymphoid enhancer-binding factor 1 antisense RNA 1
SOX9: SRY-Box 9
CPEB4: Cytoplasmic polyadenylation element binding proteins 4
PTN: Pleiotrophin
FOSL2: FOS-like antigen 2
LPAATβ: Lysophosphatidic acid acyltransferase
OIP5-AS1: OIP5 antisense RNA 1
FAM83H-AS1: Family with sequence similarity 83 member H antisense RNA 1
SBF2-AS1: SET-binding factor 2 antisense RNA 1
MBNL3: Muscleblind-like 3
NPC: Nasopharyngeal carcinoma
PTPRG-AS1: Protein tyrosine phosphatase receptor gamma antisense RNA 1
PRC1: Protein regulator of cytokinesis 1
TCF4: Transcription Factor-4
CUL4B: Cullin 4B
TTN-AS1: Titin-antisense RNA1
SOX21-AS1: SOX21 antisense RNA 1
ZNF385D-AS2: Zinc finger protein 385D antisense RNA 2
HIF1A-AS2: Hypoxia-inducible factor 1α antisense RNA 2
PCNA: Proliferating cell nuclear antigen
FBXW7: F-box with 7 tandem WD40
ROCK2: Rho-associated protein kinase 2
LIFR-AS1: Leukemia inhibitory factor receptor antisense RNA 1
